# Aortic valve disorders and left ventricular assist devices

**DOI:** 10.3389/fcvm.2023.1098348

**Published:** 2023-02-23

**Authors:** Deepak Acharya, Toshinobu Kazui, Dina Al Rameni, Tushar Acharya, Edward Betterton, Elizabeth Juneman, Renzo Loyaga-Rendon, Kapildeo Lotun, Ranjith Shetty, Arka Chatterjee

**Affiliations:** ^1^Division of Cardiovascular Diseases, University of Arizona, Tucson, AZ, United States; ^2^Division of Cardiovascular Surgery, University of Arizona, Tucson, AZ, United States; ^3^Artificial Heart Program, University of Arizona, Tucson, AZ, United States; ^4^Cardiovascular Diseases, Spectrum Health, Grand Rapids, MI, United States; ^5^Division of Cardiology, Carondelet Medical Center, Tucson, AZ, United States

**Keywords:** advanced heart failure, left ventricular assist device, aortic valve, aortic insufficiency, transcatheter aortic valve replacement

## Abstract

Aortic valve disorders are important considerations in advanced heart failure patients being evaluated for left ventricular assist devices (LVAD) and those on LVAD support. Aortic insufficiency (AI) can be present prior to LVAD implantation or develop *de novo* during LVAD support. It is usually a progressive disorder and can lead to impaired LVAD effectiveness and heart failure symptoms. Severe AI is associated with worsening hemodynamics, increased hospitalizations, and decreased survival in LVAD patients. Diagnosis is made with echocardiographic, device assessment, and/or catheterization studies. Standard echocardiographic criteria for AI are insufficient for accurate diagnosis of AI severity. Management of pre-existing AI includes aortic repair or replacement at the time of LVAD implant. Management of *de novo* AI on LVAD support is challenging with increased risks of repeat surgical intervention, and percutaneous techniques including transcatheter aortic valve replacement are assuming greater importance. In this manuscript, we provide a comprehensive approach to contemporary diagnosis and management of aortic valve disorders in the setting of LVAD therapy.

## Introduction

Left ventricular assist device (LVAD) physiology has important effects on aortic valve (AV) structure and function. AV disorders, particularly aortic insufficiency (AI), can impair the efficacy of LVAD support. AI is either present prior to LVAD implantation or develops *de novo* during LVAD support. The management of AI is challenging, and its occurrence can lead to persistent heart failure symptoms after LVAD implantation, with significant morbidity and mortality. We provide a comprehensive review of the epidemiology, pathophysiology, clinical evaluation, prevention, and management of AV disease in patients being considered for LVAD therapy and those on LVAD support.

## Aortic insufficiency

### Epidemiology of aortic insufficiency in LVAD patients

The importance of AI during LVAD support and the need for appropriate management was understood during the early days of LVAD support with pulsatile flow devices (1, 2). As the number of patients with durable LVADs for long-term support increased and as continuous-flow (*CF*) durable LVADs became mainstream, the impact of AI on VAD function and clinical outcomes became increasingly recognized (3). AI can be present prior to LVAD implantation or develop in a previously competent AV (*de novo* AI). In an early retrospective single center study, echocardiograms of 78 patients with Heartmate XVE and Heartmate II LVADs without evidence of AI at the time of implant were reviewed. Freedom from moderate to severe AI was 89.4% had 6 months, 74% at 12 months, and 49% at 18 months. Predictors of progression included female sex, smaller body surface area, Heartmate II device, increasing aortic sinus diameter, and AV that remained closed or intermittently opened, and lower ventricular volumes (4). Another single-center study of 232 patients with *CF* LVADs, primarily HMII found that greater than mild *de novo* AI during LVAD support occurred in 22.4% at 1 year and at least moderate AI was expected in 37.5% at 3 years. An AV that did not open was strongly associated with AI with hazard ratio of 11.2 (5).

In an INTERMACS analysis of 10,603 patients who had no or mild AI during device implantation, 55% of patients had at least mild AI at 6 months follow-up and 14% had moderate AI at 2 years. Predictors of progression to moderate–severe AI included age > 60 years, female sex, BSA < 2.0 m2, and mild pre-implantation AI. Of patients with mild pre-implant AI, 18.9% progressed to moderate–severe AI whereas 10.7% of those with no pre-implant AI progressed to moderate–severe AI. Long support on destination therapy devices was associated with higher rates of moderate–severe AI (6).

Aortic insufficiency remains a challenging issue with current generation devices. In a single-center study of 61 patients with Heartmate 3 who had no significant AI at implant, 20% had significant AI at 3 months post-implant. These patients had a higher rate (HR 2.76) of heart failure readmissions or death compared to those without significant AI at 1 year (7) Another single-center report evaluated 121 patients who underwent HeartMate 3 implantation and 270 Heartmate II implantation with no/trace AI at baseline and who did not undergo aortic intervention at the time of LVAD implant. They concluded that at 1 year, 26.26% of the HeartMate II group had mild AI and 15.15% had greater than mild AI whereas 34.55% of the HeartMate 3 group had mild AI and 7.27% had more than mild AI. Multivariable analysis showed no difference in *de novo* AI development between HeartMate II and HeartMate 3 (*p* = 0.68) (8) In a large single-center analysis of 836 LVAD patients with 6 year follow-up, progression to moderate or severe aortic insufficiency was lower in the HeartMate 3 group than HeartMate II groups (9.92 vs. 17.04%, *p* = 0.01). Multivariable analysis showed a signal toward less progression to moderate/severe AI in HeartMate 3 (HR 0.62, *p* = 0.053). The rate of progression was not different in the two groups in year one post implant, with HeartMate 3 having lower rates of AI progression after year 1 (9). Preliminary analysis from the MOMENTUM trial suggested lower rates of clinically significant AI in the HeartMate 3 than HeartMate II group (5.6 vs. 11.5%, *p* < 0.01) at 2 years, with further analysis ongoing (10).

### Pathophysiology of aortic insufficiency in LVAD patients

The pathophysiology of AI on LVAD support is complex. The patterns of hemodynamic stress on the AV and root are altered with LVADs. If the total cardiac output is coming predominantly from the LVAD, Left Ventricular (LV) wall stress decreases but the pressure load on the AV increases throughout the cardiac cycle, which leads to leads to valvular endothelial trauma and valvular deterioration (11). In addition, a persistently closed AV may result in commissural fusion (12, 13). There are structural changes in the aorta with continuous flow LVAD support, with an increase in adventitial thickness and intimal/medial collagen intensity associated with downregulation of extracellular matrix-degrading enzymes (14). The altered aortic root biomechanics can lead to aortic cusp remodeling (15). The proximal thoracic aorta can also enlarge during LVAD support, a phenomenon associated with hypertension (16). All these factors contribute to the development of LVAD–AI. The importance of AV opening has been recognized and is factored into contemporary LVAD design with intermittent speed drops to promote pulsatility and AV opening (17).

**Figure 1:**
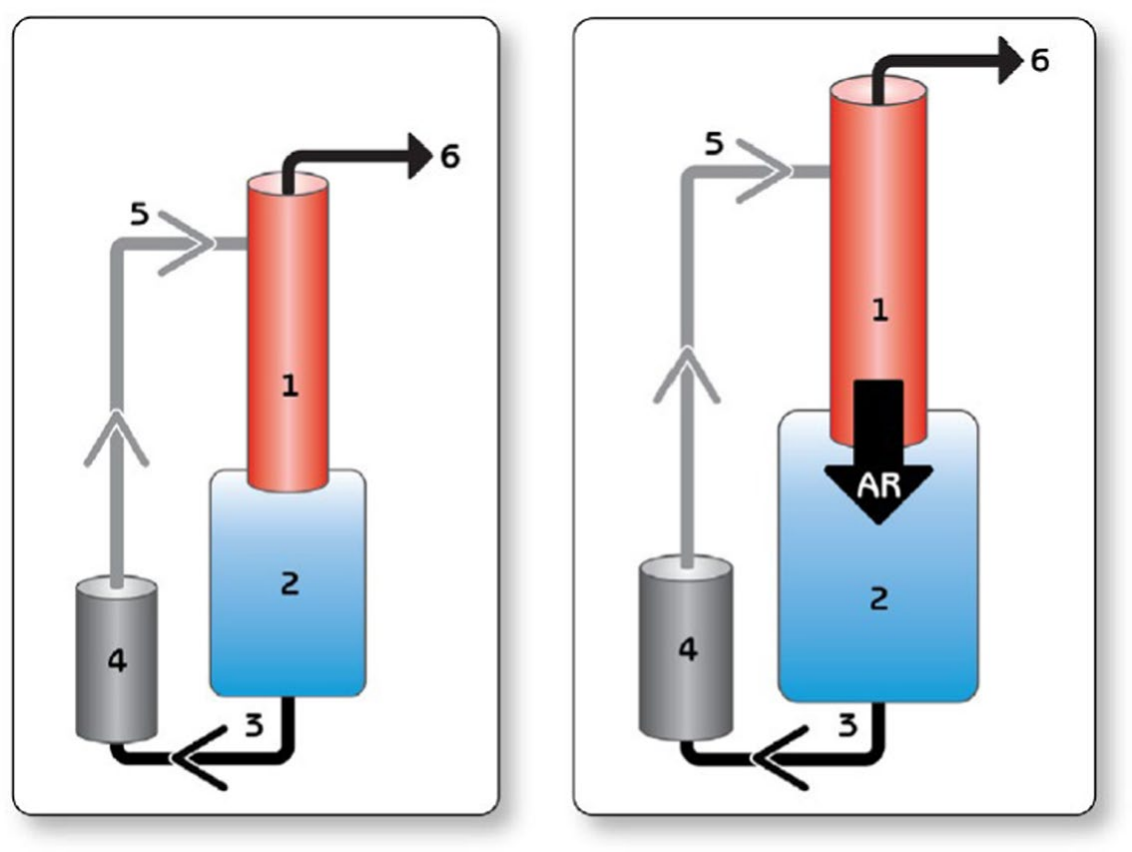
Blind circulatory loop in the setting of AR. Left, normal circuit. Right, In the setting of AI, a portion of the LVAD output regurgitates through the AV into the LV and back again through the LVAD, creating a blind loop and decreasing the effective forward flow and, hence, end organ perfusion. 1 = aorta; 2 = left ventricle; 3 = inflow cannula; 4 = pump; 5 = outflow cannula; and 6 = peripheral perfusion. AR, aortic regurgitation. Reproduced with permission from (12).

The hemodynamic consequences of AI are manifold. The cycle of blood from LVAD to the aorta, then retrogradely to the LV leads to inadequate forward cardiac output despite normal or high LVAD flows (Figure 1). LV dimensions can increase from the higher LV volume, which can predispose to mitral regurgitation. Inadequate LV offloading and increased MR can lead to elevated wedge pressure, pulmonary venous hypertension, and persistent RV dysfunction. As a result, patients can develop persistent heart failure symptoms, impaired tissue perfusion, and volume overload. This can lead to a persistent cycle of worsening heart failure and also diminish any likelihood of myocardial recovery.

### Clinical evaluation of aortic insufficiency in LVAD patients

#### History and physical exam

Mild to moderate LVAD AI may be asymptomatic, at least initially. However, with increasing severity of AI, patients may have persistent or recurrent heart failure symptoms, with dyspnea, exertional intolerance, orthopnea, and abdominal or leg swelling. Diuresis may be required, and if the patient is already on diuretics, then dose adjustment might be needed. Both left and right-sided heart failure symptoms may present as significant AI can lead to worsening RV function. Physical exam may reveal JVD, abdominal or peripheral edema. AI murmur may not be heard over LVAD sounds (18). The classical physical exam signs of AI (e.g., Corrigan’s pulse, Water hammer pulse etc.) are not present given continuous flow physiology.

#### LVAD device changes

Aortic insufficiency generally worsen over time with continuous flow LVADs (3). With both the Heartware and HeartMate platforms there is device data that can be trended to give the clinician an insight into the status of the valve.

**Figure 2:**
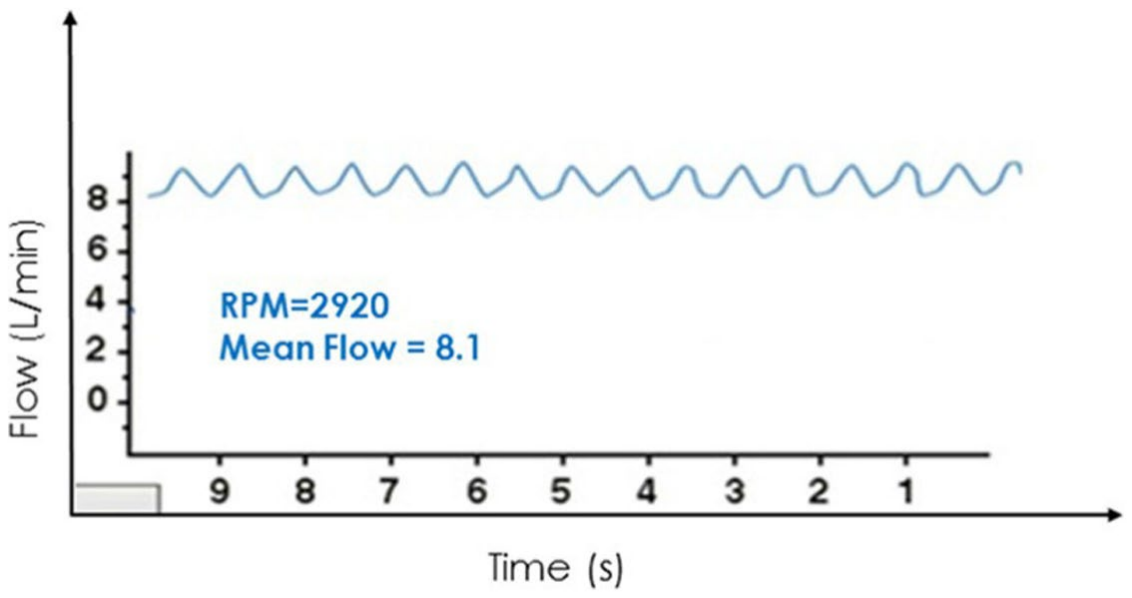
Representative heartware tracings of severe aortic insufficiency. Reproduced with permission from (19).

All *CF* VAD operations are impacted by the change in pressure differential across the pump. This principle of operation has been well documented in the development of the HeartWare waveform or delta P across the pump (19). During periods of worsening AI this pressure differential equalizes (aortic pressure verses left ventricle pressure) and throughout the cardiac cycle there is increased intraventricular volume. This leads to decreased pressure gradients across the pump and reported higher cardiac output. This reported high output is due to the creation of an alternative flow pattern of blood recycling through the AV and not forward flow to the patient (Figure 2). For Heartmate II and HeartMate 3, there is no real time graphical representation of this phenomena, but there are two key values that can be trended over time. These are estimated cardiac output and pulse index. The pulse index calculated as follows:


$$\frac{\left(PowerMax-PowerMin\right)}{\left(PowerAvg\right)}$$

This change in pump power over time is an attempt to illustrate the power variability during the cardiac cycle (systolic verses diastolic). Just like with Heartware, during AI the pressure gradient narrows, and the trough rises. This will lead to increasing reported values of flow and conversely a reduction in PI values. This reduction in PI is due to the numerator in the equation decreasing with the pressure narrowing throughout the entire cardiac cycle. Therefore, AI should be considered a patient with clinical signs and symptoms of persistent heart failure who has high flow and low PI (20).

#### Echocardiogram

##### Echocardiographic evaluation of the patient before LVAD implantation

Echocardiography is essential in assessing pre-implantation bi-ventricular size and function and ruling out valvular conditions like mitral stenosis and AI, which may reduce LVAD inflow or compromise forward flow by endless loop formation, respectively (21). Gauging pre-implantation AI severity is critical as it typically worsens post LVAD. Parameters like regurgitant jet width to Left Ventricular Outflow Tract (LVOT) diameter ratio, vena contracta, and proximal flow convergence that rely on color Doppler imaging may perform sub-optimally in severe heart failure due to low trans-aortic gradients from low mean arterial pressure and systemic vascular resistance and elevated LV diastolic pressure (21, 22). Pressure half-time can also be shortened by high LV filling pressures (22). A comprehensive evaluation using multiple different parameters is therefore needed. Size and structure of the aortic root and cusps should be carefully reported as aortic root enlargement and leaflet sclerosis/fusion may be clues to incompletely imaged eccentric regurgitation jets. Transesophageal echocardiography (TEE) can sometimes help with better visualization. In cases of doubt, phase-contrast cardiac magnetic resonance (CMR) imaging through the aortic root can provide a more volumetric assessment (22).

##### Echocardiographic evaluation of the patient after LVAD implantation

Left ventricular assist device reverses trans-aortic pressure gradients. Continuous flow from LV apex to the ascending aorta decreases LV pressure and increased aortic pressures, worsening AI duration, and severity. Remodeling of the aortic apparatus from cusp fusion and aortic root dilatation also contribute to a larger regurgitant orifice area (23, 24).

Surveillance post-LVAD echocardiograms are generally recommended at 2 weeks, 1, 3, 6, and 12 months and subsequently at 6–12-month intervals (21). AI should be evaluated at each exam.

Aortic valve opening should periodically be assessed since a closed AV is more likely to undergo commissural fusion and cusp deterioration. M-mode can be useful for measuring the frequency of valve opening and degree and duration of cusp separation. Five-six cardiac cycles at sweep speeds of 25–50 mm/s should be evaluated. Depending on LV contractility and LVAD pump speed, the AV can open with every beat, intermittently, or not at all. High pump speeds reduce AV opening. Ideally, AV should open at-least intermittently and for >200 ms as measured by M-mode (21).

As with native anatomy, a vena contracta width >3 mm and jet width to LVOT ratio >46% should represent at least moderate AI in the setting of LVAD (21). However, AI from LVAD may extend variably into the systolic phase and can even be present throughout the cardiac cycle. This phenomenon of holo-cyclic AI from LVAD induced reversed aortic gradients may not be fully captured by traditional measures for diastolic AI quantification. Further, the jet width may change between systole and diastole and may increase at higher pump speed. At high pumps speeds, continuous wave Doppler though the AV from a five-chamber view may detect holo-systolic and holo-diastolic AI with no forward flow. Color M-mode from a parasternal long axis view can also detect the temporality of AI. Due to non-confinement of AI to diastole and due to dependence on loading conditions, neither pressure half time nor aortic flow reversal can be used for AI quantification with LVAD.

**Figure 3:**
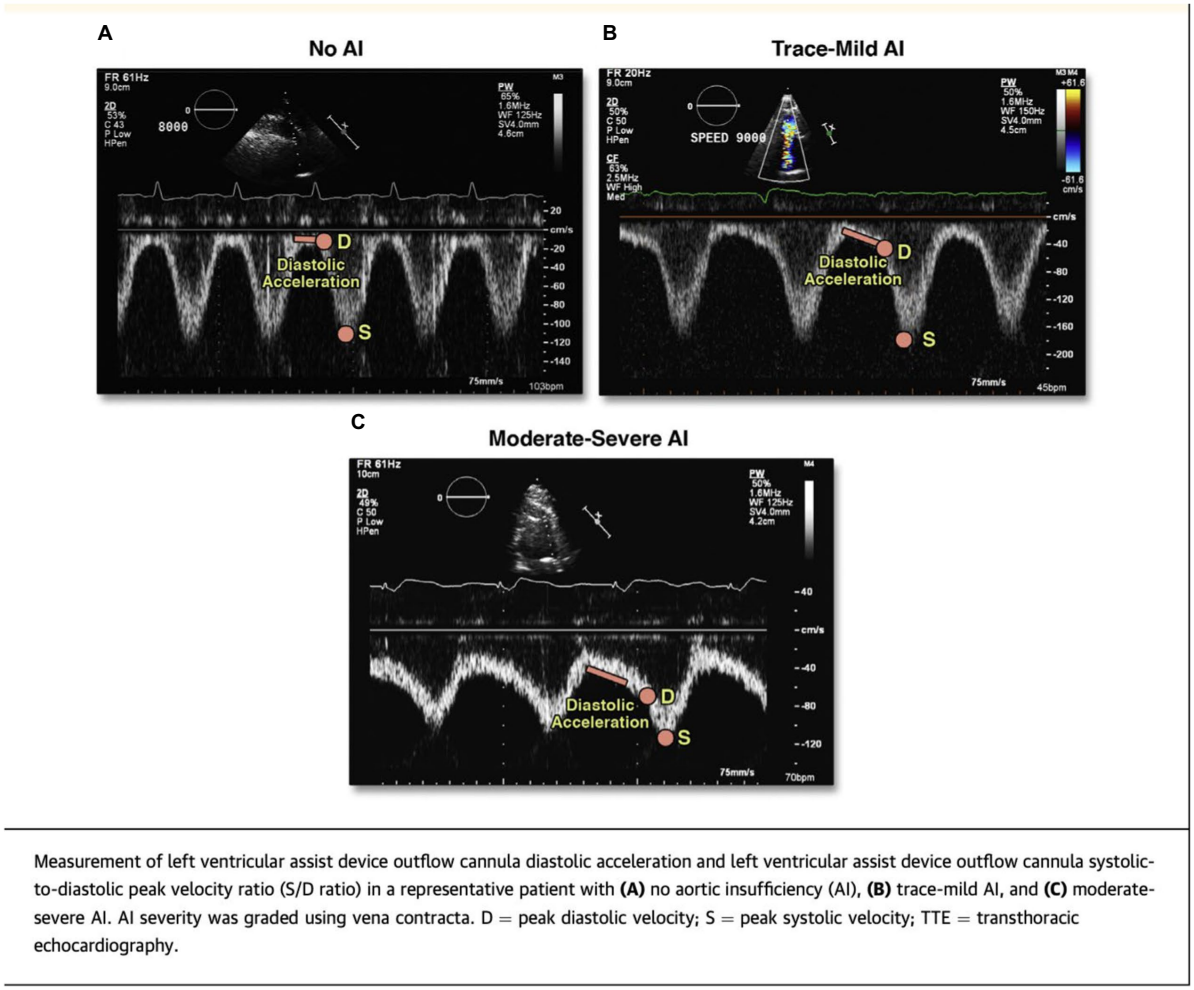
Novel echocardiographic parameters for assessment of LVAD-AI. Reproduced with permission from (25).

These difficulties have led to the evaluation of two novel echocardiographic parameters for grading AI severity with LVAD. Diastolic acceleration (dv/dt) and systolic-to-diastolic peak velocity ratio (S/D) derived from pulse wave Doppler of the LVAD outflow canula have shown better correlation with semi-invasively calculated regurgitant volume and invasive filling pressures when compared to traditional parameters like vena contracta (25) (Figure 3). These measurements are based on the augmentation of outflow cannula flow in diastole due to decreased afterload in the aorta and increased preload in the LV as seen with significant aortic insufficiency. Diastolic acceleration >49 cm/s^2^ and S/D ratio of <5.0 correlate with moderate–severe AI. These parameters can reclassify up to a third of patients with mild AI to a moderate–severe range and are able to better predict heart failure hospitalizations, AV intervention, urgent transplant, and death more accurately than vena contracta (26).

Severe AI can increase LV dimension and shift the interventricular septum to the right. Inflow and outflow cannula flows can be increased due to loop formation while Right Ventricular Outflow Tract Velocity Time Integral (RVOT-VTI) is reduced due to true reduction in cardiac output. AV interventions may typically be needed in such severe cases.

In the absence of a true gold standard for AI quantification with LVAD, a combination of parameters should be used, and interpretations should be made cautiously to avoid underestimation (21).

#### Cardiac catheterization

Cardiac catheterization has an important role in defining the severity of AI and the consequence of AI on hemodynamics and symptoms. Dynamic studies with LVAD speed adjustment, afterload reducing medications, or during exercise provide additional information (27).

The pulmonary capillary wedge pressure (PCWP) evaluates efficacy of LV offloading. It can be elevated in AI but also with other conditions, such as mitral regurgitation, severe hypertension, and inadequate LVAD speed. The right atrial pressure (RAP) reflects right ventricular (RV) function, and the relation of RAP with pulmonary artery pressure (PAP) and PCWP in can be helpful in the determination of the influence of left-sided factors on RV function. In patients with VAD-AI and no native LV ejection, the difference between the LVAD flows and cardiac output measured by right heart catheterization provides an estimate of AI volume.

Hemodynamic ramp studies can be performed during right heart catheterization and sometimes with simultaneous echocardiographic measurements. Increase in LVAD speed leads to increase in LVAD flow. However, with increased LVAD flow, LV systolic pressure can decrease, and the AV to LV gradient can increase, and can worsen AI. In one study of 55 LVAD patients who underwent simultaneous hemodynamic and echocardiographic ramp studies, the cohort with at least mild AI, ramp study with increases in LVAD speed decreased the PCWP and increased the CO, but also led to worsening AI in 78% by echocardiogram (28). This response may vary individually, and other groups have reported persistently high PCWP, lack of decrease in LV dimensions, and persistently low cardiac index despite higher pump speeds (29). Given these patients’ generally severe LV dysfunction, decreases in LVAD speed may not always improve hemodynamics and can lead to lower cardiac output and increased mitral regurgitation. Therefore, individualized assessment and adjustment to obtain the most optimal hemodynamics is important, as is recognizing that hemodynamic changes with resting ramp studies may not necessarily translate into improved exercise hemodynamics and functional capacity.

Aortogram, while not commonly performed, can be used to evaluate angiographic AI severity, aortic size, location of outflow graft, AV opening, and presence of aortic root thrombus (30).

#### Computed tomography

Computed tomography (CT) does not currently have a primary role in the assessment of LVAD AI, but provides important pathophysiological insights into many LVAD complications, including LVAD AI development and progression (31). A larger angle of the outflow graft to the aorta may direct more LVAD flow towards the AV and is correlated with AI (32).

Computational fluid dynamics using CT-derived aorta models have shown increased leaflet tip shear stress but no difference in oscillatory wall stress in those with LVAD AI relative to those without (33). Patients with AI have smaller distance from the aortic root to the outflow graft, and greater regional wall shear stress (34). Patients with AI have a perpendicular ascending aortic anastomosis (35).

### Management of aortic insufficiency in LVAD

#### Surgical management of AI at the time of LVAD implantation

Current guidelines roots for AV intervention at the time of LVAD implantation for any insufficiency greater than mild on TEE (36–38). The modality of intervention, however, continues to be a topic of debate. Concomitant procedures are associated with increased short-term morbidity, and surgeon experience and preference often dictate the surgical plan in the absence of definitive data on superiority of a particular approach (39, 40). Techniques for addressing AI support include AV closure, AV repair, AV replacement, coaptation stitch, and annuloplasty, all of which typically require cardiopulmonary bypass (CPB) and aortic cross clamping (41, 42). Technical aspects of these procedures are discussed first, followed by outcome data.

#### Aortic valve replacement

Aortic insufficiency can be addressed with a conventional AV replacement with bioprosthetic valve. The bioprosthetic valve leaflets, however, can degenerate over time and develop fuse altogether with the subsequent need for an additional future intervention.

#### Park’s stitch

**Figure 4:**
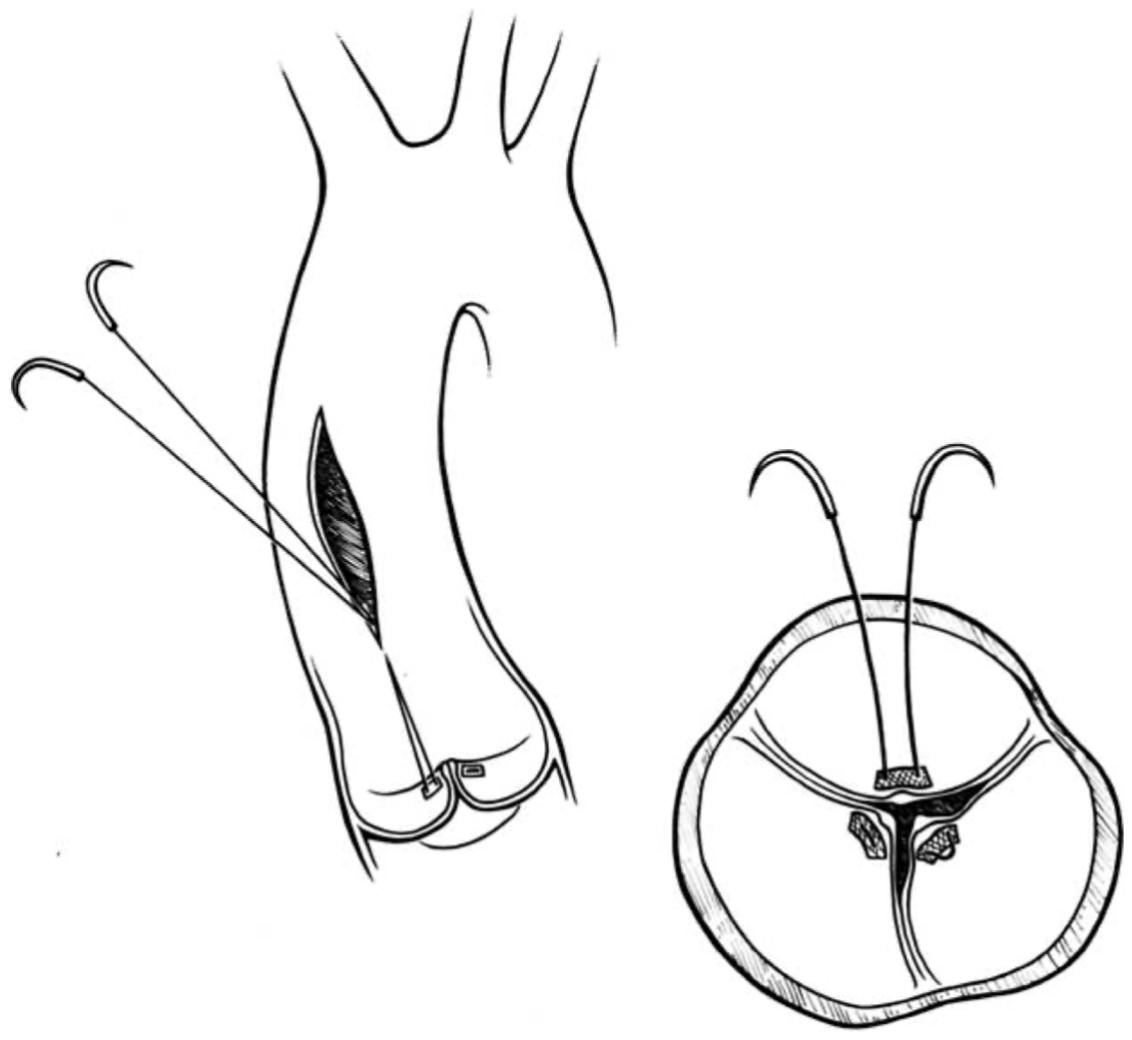
Park’s stitch. Pledgeted 4-0 Prolene sutures are applied to approximate the fibrous nodules of Arantius to create a coaptation stitch. Reproduced with permission from (1).

**Figure 5:**
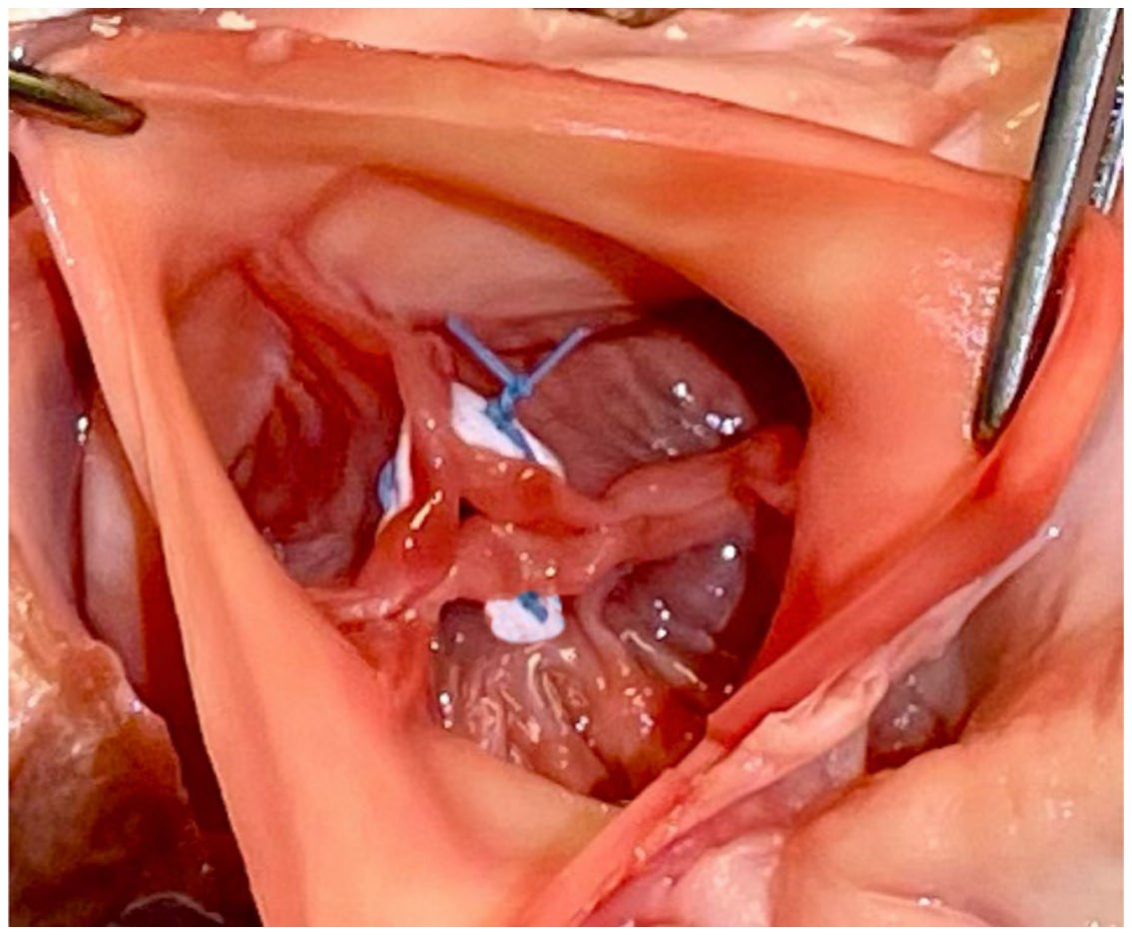
Modified park’s stitch.

In this technique, pledgeted 4-0 Prolene sutures approximate the fibrous nodules of Arantius creating a coaptation stitch. This approach allows the AV to still open for ejection, even though the effective orifice area of AV is markedly diminished (1) (Figures 4, 5).

#### Aortic valve closure

**Figure 6:**
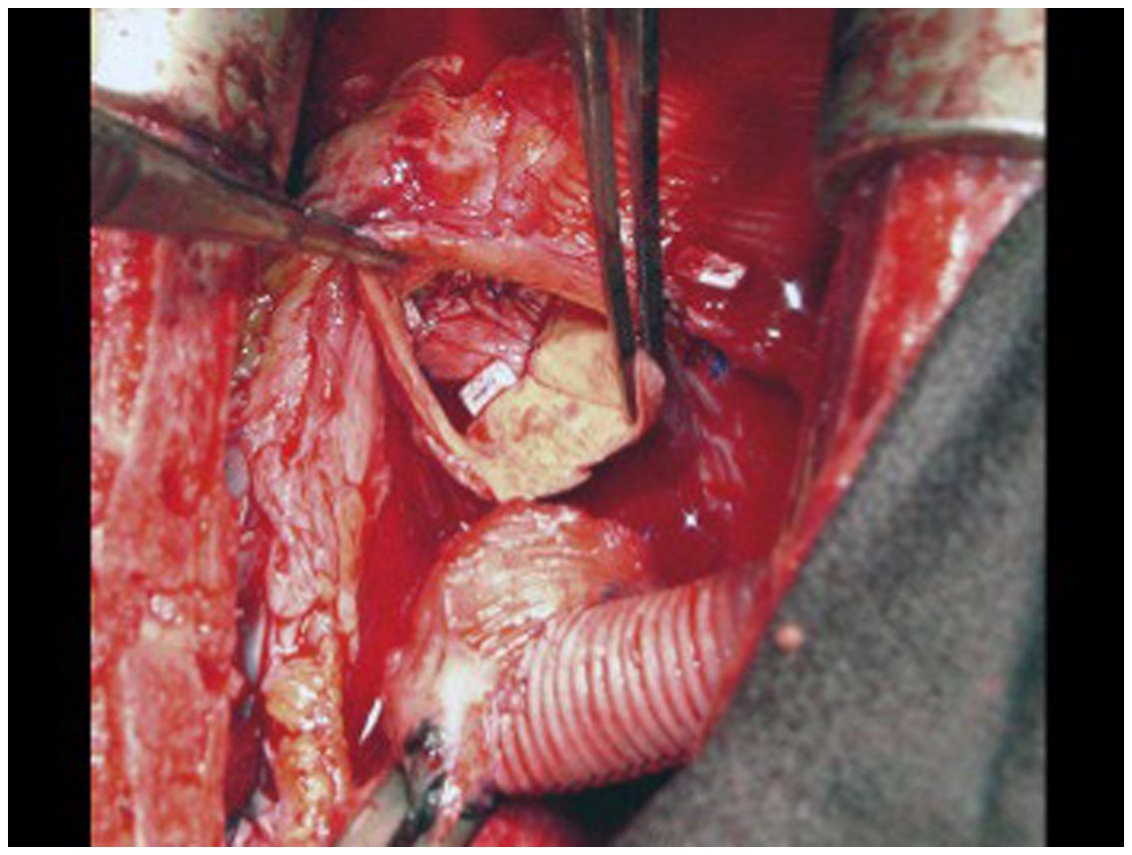
A circular patch of bovine pericardium was sutured circumferentially to the aortic annulus above the native aortic valve leaflets. Reproduced with permission from (43).

Several methods of AV closure exist. A circular patch of bovine pericardium can be sewn circumferentially to the aortic annulus above the AV, closing the LVOT (Figure 6). If there is a prior aortic bio-prosthesis, running stitches with or without pledgets along three lines of coaptation can be used to close the leaflets. For a bicuspid AV, the thickened edges of the leaflets are sewn together or a central stich in the middle of the leaflets can be placed. If there is a previous mechanical valve, it can be removed and a pericardial patch sewn circumferentially in two layers to the AV annulus (44). In the setting of a previous mechanical AV, the mechanical valve is removed, and the pericardial patch is sewn circumferentially in two layers to the AV annulus with a running 3.0 polypropylene suture (43).

#### Aortic annuloplasty

Aortic valve repair with an annuloplasty ring sutured under the valve annulus in conjunction with noncoronary leaflet plication has been successfully performed with trivial postoperative AI in a patient with HeartMate 3 intended for destination therapy (45).

#### Outcomes of concomitant AV intervention

Several single-center studies showed conflicting results on outcomes of AV interventions in LVAD candidates (42, 46). In an INTERMACS analysis of 5,344 patients who underwent LVAD implant between 2006 and 2012, 305 underwent concomitant aortic valve intervention, with 125 AV closures, 95 repairs, and 85 replacements. One-year survival was 81% for patients without AV procedure, 79% in the AV repair group, 72% in the AV replacement group, and 64% with AV closure (*p* = 0.0003). Mortality curves diverged in the first 3 months postoperatively. AV closure was independently associated with increased hazard of death in multivariable analysis (HR 1.87, *p* < 0.0001), and the most common causes of death in AV closure groups were bleeding and respiratory failure. Intervention did not guarantee success, and by 6–12 months postoperatively, moderate–severe AI occurred in 18% with AV repair, 9% with AV replacement, and 5% with AV closure.

In a recent analysis of 15,267 patients from the IMACS registry implanted with LVADs from 2013 to 2017, 457 underwent concomitant AV replacement and 328 underwent concomitant AV repair. The specifics of the repair technique were not available. Early (90 day) survival rates were 90.4% in patients without AV procedure, 85% in those with AV replacement, and 87.4% in patients with AV repair (*p* < 0.001). Late survival rates were also different (62.4, 55.5, and 60.9% in the no AV procedure, replacement, and repair respectively, *p* < 0.001). Concomitant AV replacement was an independent predictor for both early and late mortality. Mechanical AV replacement was associated with the worst outcomes.

Interestingly, those who had moderate–severe AI pre-implant, the subset that underwent no AV intervention had similar early, conditional (in 90-day survivors), and late survival to those who underwent AV repair or replacement. This led the authors to advise caution and use stringent criteria for repair/replacement, particularly for those with mild AI, and consider transcatheter AV therapies in selected cases (47).

Best practices concerning cases of mild AI at the time of LVAD need further study. With regard to surgical decision making, AV repair may be reasonable in cases of degenerative disease (cusp prolapse or malcoaptation), while bioprosthetic AV replacement could be of more value in calcific leaflet pathologies. Importantly, AV closure leaves patients completely dependent on the LVAD outflow and is, therefore, contraindicated when recovery is anticipated; furthermore, it may have catastrophic consequences in cases of pump thrombosis or malfunction. At this point, surgical intervention for mild AI may be of value if the patient has risk factors for developing *de novo* AI, such as nonischemic cardiomyopathy, an expected long duration of LVAD support (more than 1 year), and a small body surface area.

The 2013 ISHLT guidelines have no specific recommendation on preferred modality. The 2019 EACTS guidelines provide a IIa recommendation for bioprosthetic AV replacement, IIb recommendation for central coaptation stitch, and recommend against (Class III) closure of the AV (38). The 2020 AATS/ISHLT recommendations provide a Class I recommendation for addressing greater than mild AI with valve closure, repair, or replacement (37).

### How to prevent *de novo* AI at the time of initial LVAD implantation

A computational fluid dynamics study demonstrated that a closer position of the LVAD outflow graft in relation to the aortic root and angulation of outflow graft (perpendicular anastomosis to ascending aorta are risk factors for the development of *de novo* AI) (34, 35) Performing the outflow graft-ascending aortic anastomosis at a 45% angle should be considered to reduce the risk of late AI (38).

### *De novo* AI

As previously discussed, AI can develop or progress during LVAD support, with higher likelihood with longer durations of support. Therapies with some value in preventing *de novo* AI include adequate hypertension management, optimizing LVAD speeds to avoid excessive flow and persistently closed AV, and technical advancements such as intermittent pulsatility algorithms (12). Some patients with severe symptomatic AI can also be managed with intravenous inotropic therapy to enhance native contractility (48).

**Figure 7:**
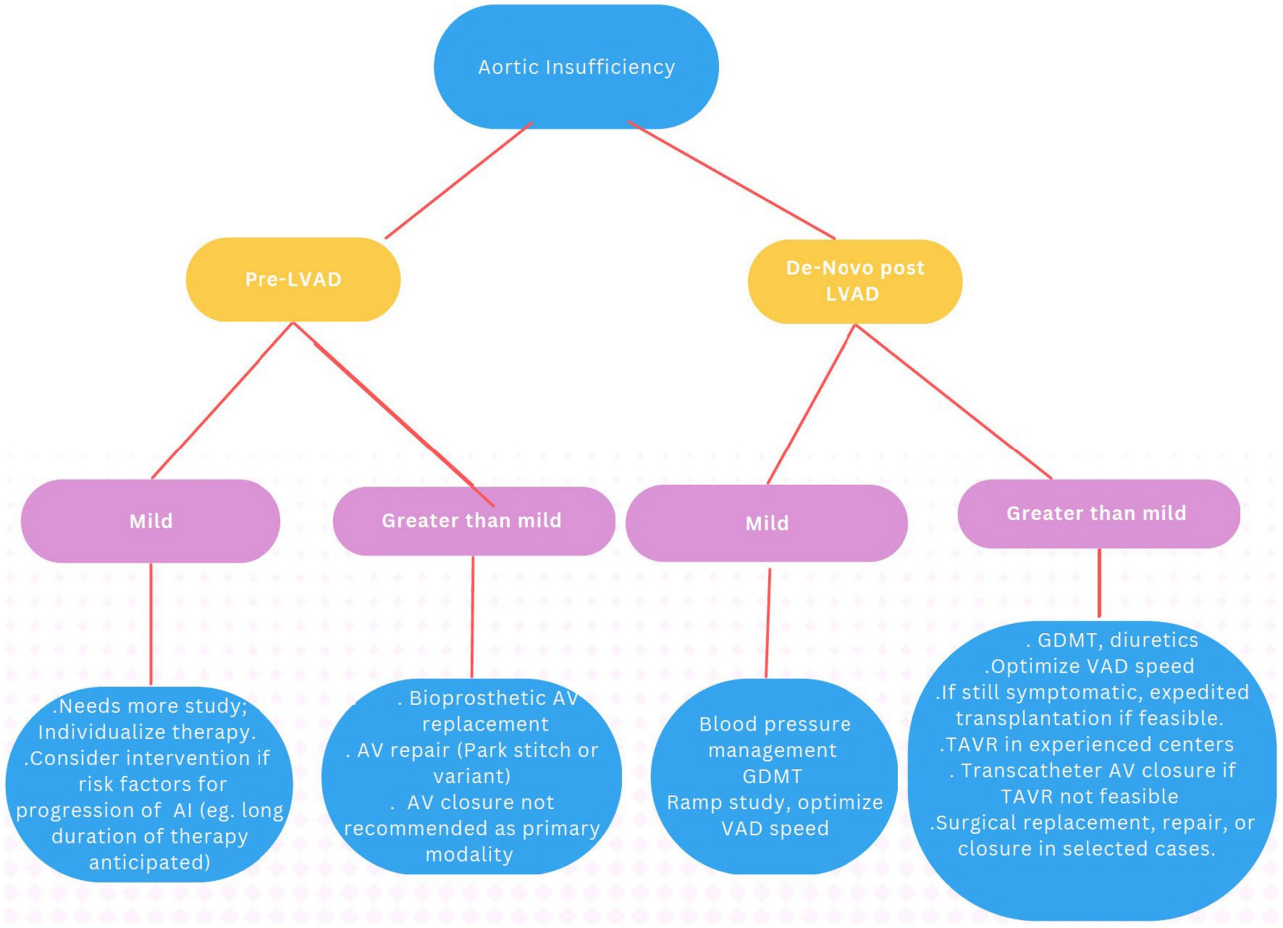
Recommendations for management of aortic insufficiency.

Most centers will consider AV intervention if patients remain symptomatic with moderate–severe AI despite medical therapy including diuretics, afterload reduction, and device optimization. The decision regarding either a surgical intervention or a percutaneous approach is made depending on the patient’s general status. Surgical intervention can be in the form of AV closure (Dacron patch), AV repair (Park stitch), AV replacement, or heart transplant (8, 49). Even though redo sternotomy on LVAD represents an invasive route with risks for RV damage, dysfunction, and significant bleeding, it remains an option in selected patients. Transcatheter therapies should also be considered alongside surgical approaches as part of an interdisciplinary approach to management. The EACTS guidelines have strong preference for heart transplant when feasible (Class I) over open valve replacement/surgical closure (Class III) for moderate AI, with transcatheter AV replacement (Class IIa) and interventional closure of AV (Class IIb) receiving intermediate recommendations. For severe AI, high urgency listing for transplantation in those who are candidates is a Class I recommendation, Transcatheter Aortic Valve Implantation (TAVI) is a Class IIa recommendation, and open valve replacement or closure and interventional closure have Class IIb recommendation (38) (Figure 7).

### Medical therapies for prevention or treatment of AI in LVAD patients

Medical therapies in LVAD patients for AI prevention or management in the current era focus on hypertension management and re-initiation of guideline-directed heart failure therapies. After normalization of cardiac output by LVAD, blood pressure, particularly diastolic BP, may increase. This may increase hemodynamic stress on the aortic root and valve and contribute to AI development. In a single-center study of 90 patients undergoing HMII and HVAD, those who developed AI had higher SBP, DPB, and MAP at three and higher DBP and MAP at 6 months than those who did not develop AI, and 3-month SBP was an independent predictor of post-LVAD AR (50). Another study of 85 patients did not find an association of BP with *de novo* AI (5). Others have shown trends implicating hypertension in AI development or progression (51). Goal MAP in society guidelines are ≤80–85 mmHg. Medications recommended are those that are standard for heart failure, i.e., ACEI/ARB/ARNI, BB, and MRAs, with the logic that these are already known to the patients, may have beneficial effects on right ventricular and renal function, afterload reduction improves LVAD functioning, the ability to use higher doses post LVAD may enhance ventricular remodeling and potential recovery (52), and the ensuing pulsatility may be helpful in AI prevention and management. The impact of SGLT2i in LVAD patients is currently not well understood but is undergoing investigation.

### Transcatheter management of AI in LVAD patients

Surgical approaches for AI management at a time later than the LVAD implant entails a reoperation in a higher-risk cohort of surgical patients and can lead to morbidity and mortality. Therefore, transcatheter therapies have assumed greater importance in recent years.

### Aortic valve closure

Aortic valve closure *via* a transcatheter approach was first reported in 2011 (53). Over the next few years, multiple reports of transcatheter AV closure were published (54–57).

The procedure is attractive because of its simplicity.

**Figure 8:**
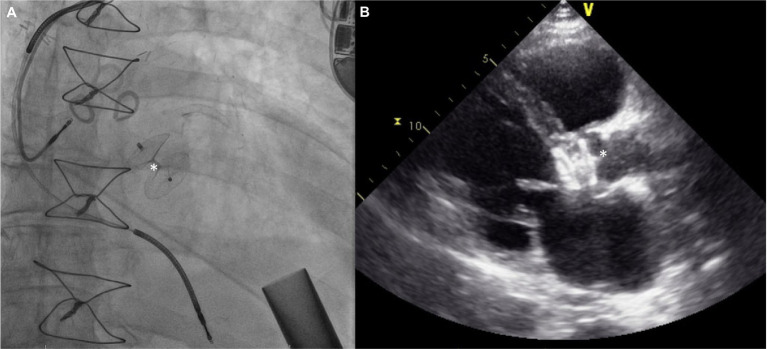
**(A)** Amplatzer device (^*^) implant for severe AI 3 years after HMII implantation. **(B)** Echocardiograpic appearance of device.

The procedural details are as follows: the AV is crossed in retrograde fashion from usually a femoral access point. Usually, a Multipurpose/Amplatz left 1/Judkins Right 4 catheter is used to cross the valve with a straight tip wire. Next, a stiff wire (Amplatz Extra stiff or a pre-formed helical tip wire, e.g., Safari or Confida wire) is placed in the LV. Over this wire, a Torqvue 45° delivery sheath is advanced across the native AV. Since the length of the Torqvue sheath is limited at 80 cm, it might be necessary in tall patients to consider alternate access or consider a longer 8F/9F sheath (Flexor). Sizing of the device is done *via* TEE or with gated multidimensional CT. Care is taken not to oversize the device beyond the size of the aortic annulus to decrease the chances of interaction with the anterior mitral leaflet or with the coronary ostia. The Amplatzer Cribriform septal occluder (CSO) is almost universally used, however the initial report used an Amplatzer post infarct muscular ventricular septal defect occluder. Post deployment, aortogram is used to ensure no coronary compromise. It is common to see unresolved AI for a short period of time until the device pores start to thrombose (Figure 8).

The patient in the first report by Grohmann et al. improved, although with hemolysis requiring transfusions for up to 6 weeks which caused renal dysfunction—this necessitated stopping anticoagulation to try and promote thrombosis of the device. The patient only survived a few more weeks although the death was related to an accident with battery exchange. At autopsy, the device was well seated and did not cause any coronary compromise (53). Parikh et al. have published the first case series of five patients (54). Amplatzer Cribriform septal occluders were used successfully in all patients. Hemodynamic improvement was noted in all patients acutely. However, there was embolization of the device to the aortic arch in one patient and two other patients did not survive to the 30-day mark despite a stable device. The embolization was thought to be a result of interaction with the struts of a pre-existing bioprosthetic mitral valve. In a systematic review with data on 21 patients, two out of 21 AV closure devices embolized, although not all series included reported on procedural complications (58). Sauer et al.’s patient survived 10 months without major complications and successfully had a transplant—the CSO device appeared to be well seated and endothelialized at explant (55). In a later analysis likely including the patients included in the series by Parikh et al., Retzer et al. compare the characteristics of 10 patients who underwent percutaneous AV closure—three survived to discharge and subsequently were alive at 6 months (59). Non-survivors were more likely to have worse kidney function and have higher pulmonary artery systolic pressure. They were also likely to have higher lactate dehydrogenase levels post implant and develop worsening RV dilation. An interesting point raised is the size of the device used compared to the aortic annulus, and patients who got smaller devices (device to annulus ratio < 0.9) were more likely to survive, suggesting a role for interaction with other cardiac structures. The major criticism of using this technique is that it renders the patient pump dependent.

### Transcatheter aortic valve replacement

Transcatheter aortic valve replacement (TAVR) has become mainstream therapy for aortic stenosis and is used off label for patients with AI in selected patients with suitable anatomy (60, 61). TAVR in AI has its challenges primarily because of lack of calcification of the AV leaflets and annulus. Annuli in AI patients often are dilated and may be beyond the specifications of valve systems. Both self-expanding and balloon expandable valves have been used for aortic insufficiency in patients with LVADs.

The procedural details are as follows: planning for TAVR in LVAD patients is approached in the usual fashion with CT to measure the annular/LVOT parameters and surrounding anatomy. Transfemoral access is most commonly used, although subclavian/axillary artery approach has also been reported.

**Figure 9:**
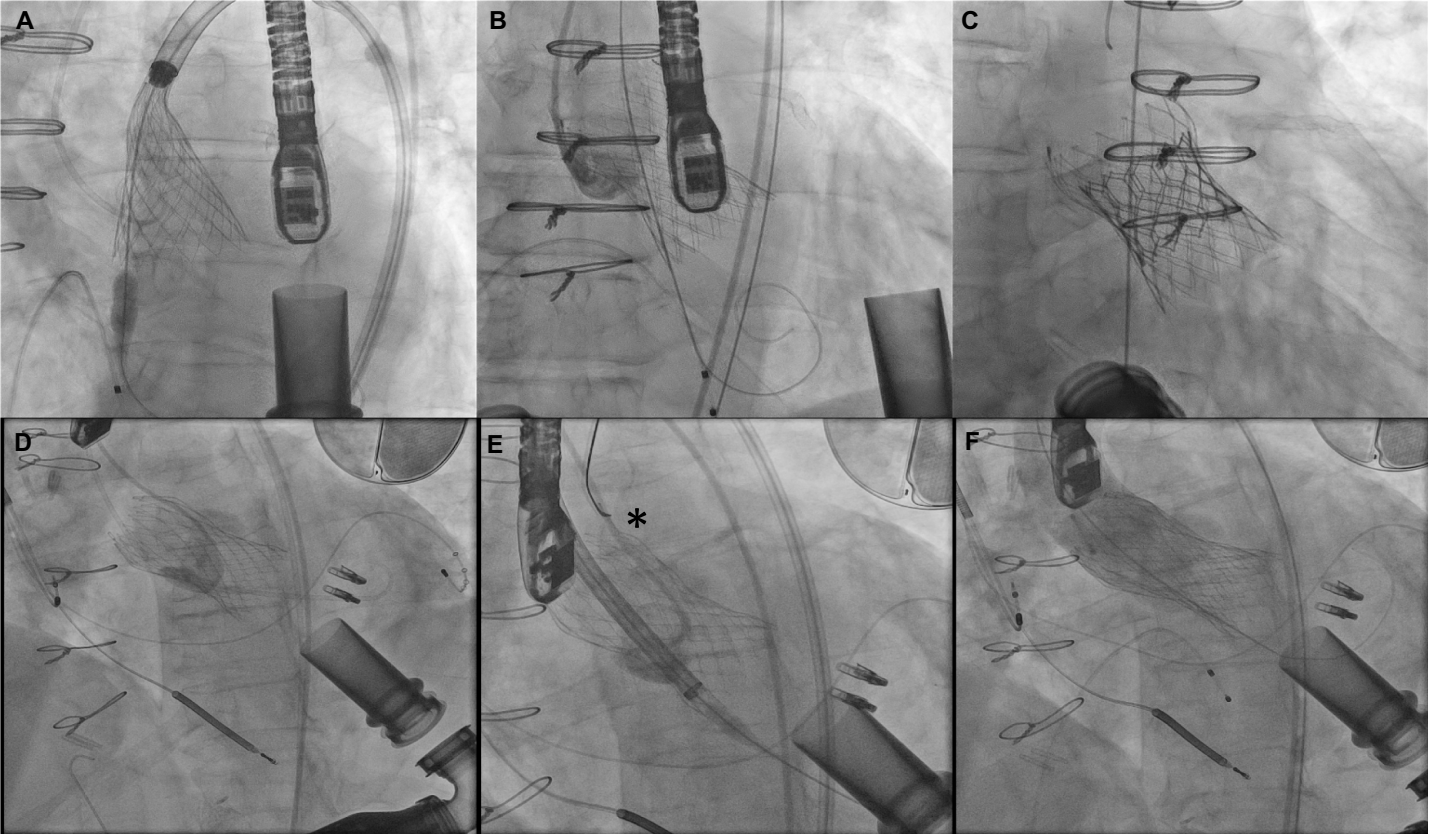
**(A–C)** Attempted Evolut R implant with ventricular migration immediately after release stabilized with 29 mm Sapien 3 implant inside the malpositioned Evolut. **(D–F)** Ventricular migration of Evolut R valve in another patient with repositioning using a gooseneck snare (asterisk) and implantation of a second Evolut R inside with post dilation reducing aortic insufficiency to trace.

More cases have been reported with use of self-expanding valves, with the older generation as well as more current valve iterations. Use of a stiff wire for delivery is generally recommended with the Lunderquist double curve wire being commonly used. Oversizing with a range close or slightly over 30% is essential. The theoretical benefit of using a self-expanding platform is being able to recapture and test the valve in a 75–80% deployed state for longer durations of time to test stability. Longer pacing runs to allow the valve to expand more and stabilize are recommended. Still, valve migrations are common, hence being prepared to stabilize/pull up the valve with a single/double snare technique is required (62). Additional 6F accesses are required for this purpose and a double snare theoretically has a greater chance of successfully repositioning the valve and reduce the risk of aortic injury since the valve frame is compressed by pulling forces on the tabs from either side. The other approach is to place a second valve, usually a balloon expandable Sapien valve using the Corevalve/Evolut as a scaffold (Figure 9). However, this does not work for valve frames that are extremely deep.

Less often, balloon expandable valves of the Sapien family have also been used to treat AI in LVAD patients. Oversizing is of paramount importance here as well and oversizing in excess of 20% is better tolerated than in aortic stenosis patients. Postdilation is often required, and deployment with additional volume is reported anecdotally, based on reports of ability to over-expand the Sapien 3 family of valves without losing competency (63).

The first report of TAVR to treat LVAD associated AI was made by Santini et al. in 2012 (64). In what has been seen in other series that followed, the first Core valve that was deployed was not stable and had at least moderate perivalvular leak. A second Core valve was deployed inside the first one leading to an improved outcome, albeit still with mild peri-valvular leak. More reports of TAVR for LVAD AI have used self-expanding valves. Yehya et al. have reported the largest series with 6 months follow up (65). In two of the nine patients, there was acute valve migration into the LV necessitating snaring of the valve to correct the position and deployment of a second valve (one Sapien 3, one Core valve). One patient died 4 months after TAVR. There was no significant AI in the remaining eight patients at 6 months. Four of these TAVR valves appeared completely closed. There was improvement in RV function and tricuspid regurgitation and a median NYHA II functional class was maintained. This and other early reports include TAVR with first generation of self-expanding valves. The newer iteration of the Evolut platform including a 34 mm valve is a more intuitive choice in order to achieve greater oversizing and more radial force on the non-calcified annulus. Dhillon et al. report a series of four such cases using the 34 mm Evolut valve (66). Three of their cases were uncomplicated, however the fourth had significant ventricular migration which was managed with implantation of a 29 mm Edwards Sapien 3 valve in the waist to try and post dilate/stabilize the valve. The patient continued to have mild–moderate perivalvular leak, eventually had fusion of the valve leaflets and was not able to be rescued. Two of the other three patients also did not survive to 3 months. In a retrospective multi-center study evaluating TAVR for native AI, newer generation valve systems like the Evolut had significantly less chance of having a malposition and greater than moderate leak compared to first generation Core valve (67).

Kar et al. reported three cases using the balloon expandable Sapien 3 platform—two cases with 26 mm and one with a 29 mm valve (68). There were no immediate complications and there was significant resolution of AI in all patients. One patient was transplanted >2 years post procedure, another was reported alive 1,120 days post TAVR with mild AI while the third patient died at home 616 days post TAVR with unknown cause of death. The introduction of other balloon expandable valve options has widened the scope of use in aortic insufficiency cases. Recently, a patient with LVAD associated AI was treated successfully with a 32 mm MyVal valve (69).

Beyond self-expanding and balloon expandable platforms, a leaflet anchoring new valve platform is now CE mark approved in Europe (70). The JenaValve system has three locators which anchor to the three AV cusps. There are case reports of this valve system being used for LVAD associated AI cases without any major instability or complications (71). The JenaValve transfemoral system is being studied with the ALIGN-AR trial currently. The *J*-Valve is a valve based on a similar concept with three “rings” to clasp the native leaflets. There are a wide variety of sizes available (22–34 mm) (72). Although not fully mature, with more experience, leaflet anchoring valve platforms will likely be the mainstay of treating native AV insufficiency, making decision making for LVAD associated AI simpler.

### Outcomes

Despite the pathophysiologic derangements, several early single-center studies did not show a consistent association of *CF*-LVAD-AI with worsened clinical outcomes or higher mortality. Cowger and colleagues evaluated a single-center cohort of 166 HeartMate two patients, of whom 131 were bridged to transplant. Moderate or higher AI was present in 33% of patients at 2 years but was not associated with a higher hazard of developing worsening mitral regurgitation or RV dysfunction. No survival difference was observed between those with moderate or higher degrees of AI vs. lesser degrees of AI. Only three of 35 deaths were attributed directly to AI (73). Another single-center study of 79 patients (87% DT indication) found development of mild or greater AI in 52% at a median of 187 days f/u. There were no significant differences in heart failure hospitalizations or BP in those with vs. without AI. Mortality was increased in patients with AI, and AI was a significant predictor of death (OR 3.14, *p* = 0.005) but no statistically significant difference in survival curves by log-rank test was observed (74). In a single-center study from the United Kingdom evaluating 93 patients with both HeartMate II and Heartware, longer duration of support and persistently closed AV were associated with development of AI, but no association of mild or greater AI with mortality was noted (75). Another single-center study of 210 Heartmate II patients with 79% of the cohort being bridge to transplant and median support duration 582 days, moderate or severe AI developed in 15.2%. No deaths were directly attributed to AI and there was no difference in survival in those with or without significant AI (76). Important limitations of several of these studies were small numbers of patients, single center practice pattern nuances, predominantly bridge to transplant populations with short-term follow-up with very low numbers of at-risk patients at later time-points, and lack of time-varying analyses.

In the largest published experience to date from INTERMACS, compared to patients with no/mild AI, those with moderate/severe AI hade lower systolic blood pressures, higher left ventricle end diastolic diameter, higher pro-Brain Natriuretic Peptide, and higher degree of at least moderate regurgitation. Patients who developed significant AI in the first year of device support had lower freedom of hospitalization at 2 years, without significant differences in stroke, arrhythmia, and bleeding. Most importantly, survival was also affected: those who developed moderate–severe AI had lower survival (49.1 vs. 36.5% at 5 years, *p* < 0.001) compared to those who had no-mild AI. Differences in survival persisted after adjustment for age, INTERMACS profile, and chronic kidney disease, and on a conditional analysis of 1-year survivors (6).

## Special populations

### Aortic stenosis

Significant aortic stenosis in patients with severe LV systolic dysfunction should be addressed promptly as surgical or transcatheter AV replacement may improve LV function enough to obviate the need for LVAD. Aortic balloon valvuloplasty is generally not advised other than as palliative therapy and may complicate matters if significant AI results. Aortic stenosis *per se* does not affect LVAD function. However, severe aortic stenosis may impair LV recovery and reduced aortic excursion may lead to further leaflet fusion and risk late AI. Therefore, surgical or transcatheter AV replacement should be considered on a case-by-case basis.

### Pre-existing prosthetic AV

Patients with a functioning bioprosthetic AV at the time of LVAD implant do not need additional AV intervention. Those with a degenerated bioprosthetic AV are likely best treated with another bioprosthetic AV, but evidence is scant. In general, mechanical AV should be replaced with a bioprosthetic AV at the time of LVAD implant. Closure of mechanical AV is technically feasible but is associated with poorer outcomes, renders the patient completely LVAD dependent, and does not permit LV recovery, and is therefore not recommended as a first line therapy. Another technique recently reported involves breaking the inner leaflets of the mechanical AV and sewing a bioprosthetic valve on top of the mechanical valve ring (77).

### Conclusion

Aortic insufficiency is a common LVAD-associated problem. Incidence increases with duration of support and can lead to morbidity and mortality. Pathophysiology is complex and involves patient-related, medical management-related and device-related factors. Management can be challenging and incorporates medical, device engineering, percutaneous, and surgical approaches. There is an unmet need for larger scale randomized studies to provide more robust evidence on optimal approaches to prevent and treat AI.

## Author contributions

DA: conception. DA, AC, DR, TA, and TK: draft. DA, TK, DR, TA, EB, EJ, RL-R, KL, RS, and AC: editing and critical revisions and final approval. All authors contributed to the article and approved the submitted version.

## Conflict of interest

The authors declare that the research was conducted in the absence of any commercial or financial relationships that could be construed as a potential conflict of interest.

## Publisher’s note

All claims expressed in this article are solely those of the authors and do not necessarily represent those of their affiliated organizations, or those of the publisher, the editors and the reviewers. Any product that may be evaluated in this article, or claim that may be made by its manufacturer, is not guaranteed or endorsed by the publisher.
